# Examining the Effects of 2SLGBTQI+ Candidates on 2SLGBTQI+ Voter Turnout in Canada

**DOI:** 10.1177/10659129251355606

**Published:** 2025-06-26

**Authors:** Joanna Everitt, Kenny William Ie, Karen Bird, Angelia Wagner, Mireille Lalancette

**Affiliations:** 1 152112 University of New Brunswick, Saint John, NB, Canada; 2 248191 McMaster University, Hamilton, ON, Canada; 3 3158 University of Alberta, Edmonton, AB, Canada; 4 14847 Université du Québec à Trois-Rivières, Trois-Rivières, QC, Canada

**Keywords:** 2SLGBTQI+ candidates, candidate voter affinity, voter turnout, Canada

## Abstract

Affinity theory suggests that 2SLGBTQI+ candidates might empower and motivate 2SLGBTQI+ voters to become more politically engaged because they demonstrate that the political system is open to queer participation and voices. However, most research has focused on high-profile 2SLGBTQI+ candidates. Little attention has been paid to the impact of local candidates, particularly in a multi-party parliamentary system. This paper explores this candidate–voter affinity by incorporating information about the 70 2SLGBTQI+ candidates who ran in the 2021 Canadian federal election into survey results of the 2021 Canadian Election Study (CES). Comparing responses of 2SLGBTQI+ voters in districts with 2SLGBTQI+ candidates to those of 2SLGBTQI+ voters who do not have such candidates to select enables us to demonstrate the impact of these affinities on voter turnout. Our results reveal positive affinities, meaning 2SLGBTQI+ individuals are indeed more likely to vote and their turnout is higher in the districts with 2SLGBTQI+ candidates.

Researchers have long recognized the importance of diversity in political decision-making bodies for policy outputs, elite representation, and democratic legitimacy.^1^ Somewhat less attention has concentrated on the symbolic impact that diverse candidates’ identities might have on the political engagement of minority voters who share those identities. In particular, few observational studies have focused on candidate–voter affinities for minority communities like 2SLGBTQI+ individuals,^2^ due in part to the limited availability of large sample sizes that include an adequate number of minority respondents. In the case of 2SLGBTQI+ individuals, it is also a reflection of the fact that, until recently, few 2SLGBTQI+ candidates have run for office (Everitt and Tremblay 2023) and few large-scale election surveys have included measures asking individuals if they identify as 2SLGBTQI+ (Egan 2025).^3^

Nonetheless, such candidate–voter affinities are worth studying. Recent research suggests that unlike other social minorities 2SLGBTQI+ voters are more likely to be actively engaged in the political process (Bowers and Whitley 2020; D’Amico 2025; Moreau et al. 2019; Turnbull-Dugarte and Townsley 2020), yet much remains to be learned about what motivates them. Much of this work comes from the U.S., where partisanship is a strong and motivating factor (Hertzog 1996). But high 2SLGBTQI+ mobilization is also found in countries with multi-party systems or where parties are less polarized around 2SLGBTQI+ rights (Turnbull-Dugarte 2020a, 2020b, 2020c). Many scholars attribute this heightened involvement to an oppositional consciousness and the need of 2SLGBTQI+ voters to protect vulnerable policy gains (Turnbull-Dugarte and Townsley 2020). This raises questions about the degree to which 2SLGBTQI+ candidates can further mobilize 2SLGBTQI+ voters to turn out. We explore this question by focusing on Canada, a country with a comparatively high level of acceptance of 2SLGBTQI+ people and an even higher level of entrenched rights for them. In concentrating on Canada, we can test the impact of candidate–voter affinities in a political system where 2SLGBTQI+ voters are less likely to feel politically threatened than they might elsewhere.^4^ Despite recent backsliding, Canada ranks among the highest countries in the world on the LGBT Equality Index (equaldex.com/equality-index). By the 2021 federal election, big policy gains such as protection from discrimination based on sexual orientation, gender identity, and gender expression had been secured along with rights to same-sex marriage, survivor benefits, age of consent, adoption rights, and the ability to use an “X” descriptor on passports and other legal documents (Everitt and Wagner 2025). This presents a situation in which the 2021 Canadian election is least likely to mobilize voters in different regions or in support of different parties for oppositional reasons and enables us to more clearly isolate the impact of the presence of 2SLGBTQI+ candidates on 2SLGBTQI+ voter turnout.

We do this by incorporating information about the 70 2SLGBTQI+ candidates who ran in the 2021 Canadian federal election into the survey results of the 2021 Canadian Election Study (CES).^5^ This permits us to compare the responses of 2SLGBTQI+ voters in districts with 2SLGBTQI+ candidates to those of 2SLGBTQI+ voters who do not have such candidates to select. We are thus able to assess to what degree voters, who see candidates with whom they share their identities as 2SLGBTQI+ individuals, are more likely to turn out to vote (or not) in comparison to those voters without such local options. Our data analysis reveals strong affinity affects such that the presence of 2SLGBTQI+ candidates in a district is highly correlated to increased local turnout among 2SLGBTQI+ voters, while districts without these candidates see little difference in turnout between 2SLGBTQI+ and non-2SLGBTQI+ voters.

## Political Participation and 2SLGBTQI+ Individuals

Traditionally marginalized groups tend to be less politically engaged than those with greater social and political power. Women, Blacks, Latinos, Indigenous people, and others who have been historically disadvantaged are less likely to join political parties, donate to candidates, run for office, and engage in other non-voting forms of political participation (Arvizu and Garcia 1996; Kittilson and Schwindt-Bayer 2012; Verba et al. 1993). Interestingly, this has not been the case for 2SLGBTQI+ individuals, who have been found to be more generally politically engaged (Bowers and Whitley 2020; Flores et al. 2024; Moreau et al. 2019; Page 2018; Turnbull-Dugarte and Townsley 2020), although bureaucratic barriers might make it more difficult for transgender individual to participate than lesbian, gay, or bisexual voters (Strode and Flores 2021).

Most studies of 2SLGBTQI+ individuals, however, focus on their voting behavior more than their political participation. These studies have found that 2SLGBTQI+ voters are more inclined to vote for left-of-center parties and identify as more left-wing than non-2SLGBTQI+ voters, with variation existing among different members of the community (Hertzog, 1996; Swank, 2018; Turnbull-Dugarte, 2020a, 2020b, 2020c, 2022). The few studies on Canadian 2SLGBTQI+ voters have demonstrated similar results (Albaugh et al. 2024; Guntermann and Beauvais 2022; Perrella et al. 2012, 2019).

Much of the research on the political participation of 2SLGBTQI+ individuals point to higher turnout rates among 2SLGBTQI+ voters than non-2SLGBTQI+ voters in the United States (Egan et al. 2008) and Western Europe (Turnbull-Dugarte and Townsley 2020). Ayoub and Page (2020), however, find that turnout is suppressed in countries hostile to LGBTQ+ people. Despite a large body of Canadian research on 2SLGBTQI+ candidates, elected representatives, party leaders, and voters (Everitt and Camp 2009; Everitt et al. 2019; Everitt and Raney 2019; Everitt and Tremblay 2023; Lapointe et al. 2024; Tremblay 2022; Wagner 2021), few studies have examined the participation rates of members of the 2SLGBTQI+ community in Canada (Daoust et al. 2024; Perrella et al. 2012, 2019; Çakır et al. 2023). Perrella et al.’s (2019) analysis of the Canadian General Social Surveys of 2008 and 2013 found that lesbian, gay, and bisexual voters were more politically active than other respondents except in their propensity to vote, where no difference was found in their responses. However, Çakır and his colleagues’ (2023) analyses of data from the 2021 Canadian election study and six recent provincial elections found that similar to elsewhere, sexual minorities were more likely to report they voted in elections than other Canadians (Çakır et al. 2023) and were more politically engaged (Daoust et al. 2024).

## Theories Accounting for Political Engagement

Explanations for higher levels of political engagement can be linked to theories of social identity, linked fate, group efficacy, and oppositional consciousness. *Social identity* is the idea that a person’s identity is structured by their group memberships, which can provide important sources of pride and self-esteem (Tajfel 1979). Those who share this identity are part of one’s in-group and those who do not are considered out-group members. *Linked fate* takes that feeling of closeness to one’s in-group a step further by incorporating the notion that one’s life chances “are inextricably tied to the group as a whole” (Simien 2005, 529).

When this sense of linked fate occurs among politically empowered groups, it might result *in group or collective efficacy,* defined as a sense that members of one’s in-group can achieve desired outcomes that benefit that group (Lee 2010, p. 393). This collective efficacy is often possessed by those among the hegemonic elite in a society (i.e., those from European Christian backgrounds in Canada), but might also be found among those who have successfully experienced policy or social changes benefitting their group and who thus feel more integrated in the larger community. Such efficacy might lead them to be more politically engaged and feel more empowered to vote. Studies examining the role of race in shaping political efficacy suggest living in areas with Black mayors (Bobo and Gilliam 1990), witnessing changes to policies benefiting Blacks (Baretto et al. 2018), or having Latina/o representation in legislatures (Pantoja and Segura 2003) can enhance these groups’ levels of efficacy.

Most scholars argue, however, that the impact of linked fate is likely to be strongest when a group has been historically oppressed or marginalized. Rather than efficacy and empowerment, the resulting feeling of social exclusion and oppression leads to the development of an *oppositional consciousness* and increased political mobilization (Bowers and Whitley 2020). Mansbridge and Morris (2001) state that “Oppositional consciousness…is an empowering mental state that prepares members of an oppressed group to act to undermine, reform, or overthrow a system of human domination. It is usually fueled by righteous anger over injustices done to the group and prompted by personal indignities and harms suffered through one’s group membership” (pp. 4–5).

Collective efficacy arguments have yet to be employed to account for 2SLGBTQI+ participation, but oppositional consciousness arguments have been tested, particularly in countries where 2SLGBTQI+ rights are still contested. For example, Turnbull-Dugarte and Townsley (2020) suggested that the “over-participation” of 2SLGBTQI+ individuals might be a way for a community that for decades faced discrimination, harassment, and social contempt to ensure that 2SLGBTQI+ rights and policies are protected from electoral forces that might otherwise seek to reduced or overturn them. Others have found that in states with high levels of political homophobia and where 2SLGBTQI+ individuals have yet to win political rights, they are less politically active and less likely to vote than others (Ayoub and Page 2020; Kamenou 2024) or to engage in more contentious forms of politics than election campaigns (D’Amico 2025).

Little research, however, examines the impact of linked fate in societies where 2SLGBTQI+ groups have faced less discrimination in recent years, have secured robust legal protections, and are not actively threatened by members of the out-group. One exception is a study of Sweden, where Grahn (2024) studied turnout in four elections held after the enactment of same-sex marriage rights. He found that despite the securing of important rights, lesbian, gay, or bisexual individuals continued to turn out at higher levels than comparable heterosexual voters and argued that the protection of minority rights remained an important mechanism for the continued mobilization of sexual minorities (Grahn 2024).

## Voter-Candidate Affinities and Linked Fate

One area that has received no attention in the literature on 2SLGBTQI+ political engagement is the degree to which turnout might be influenced by the presence of political candidates with shared identities. This idea draws on theories of in-group bias and candidate–voter affinity, theories used to understand the engagement of women, Blacks, or other under-represented groups. These theories argue that individuals are more likely to favor and develop affinities with other members of their own in-group or community even when no obvious policy reasons exist to do so (Tajfel 1981). If this is the case, when candidates who are members of historically under-represented social groups are presented as options in election campaigns, we might expect them to influence voters with similar backgrounds by increasing their engagement with the campaign (Banducci et al. 2004; Dabin et al. 2019; Rocha et al. 2010).

Motivations for this affinity can be expressive or instrumental. On the one hand, expressive motivations are driven by self-identity, in-group bias, and the desire for positive group attributions, empowerment, and self-esteem (Banducci et al. 2004; Besco 2019; Goodyear-Grant and Tolley 2019). On the other hand, instrumental motivations reflect a belief that the candidate shares their interests, concerns, and experiences and would therefore be a better representative who could advance preferred policy (Landa et al. 1995). Both motivations suggest possibilities for increased engagement.

Indeed, a growing body of literature on affinity voting has attempted to apply social identity theory to the voting processes by exploring the degree to which voters are inclined to vote for those with whom they share an identity. This might be an identity based on their sex (Goodyear-Grant and Crosskil 2011; Goodyear-Grant and Tolley 2019; Sanbonmatsu 2002), race or ethnicity (Besco 2015, 2019; Bird 2016; Bird et al. 2011), Indigeneity (Dabin et al. 2019), or sexual minority status (Everitt and Horvath 2021). On the other hand, less research exists on the impact that candidate–voter affinity might have on voters’ actual political engagement, including their willingness to vote.

Researchers who have looked at the impact of affinities on political engagement have suggested that the descriptive representation results in a sense of group efficacy or empowerment among voters who share those backgrounds. They argue that as more individuals hold positions of political leadership, members of their minority group should experience enhanced trust in government, efficacy, group pride, and participation (Banducci et al. 2004; Bobo and Gilliam 1990; Tate 1991). For example, evidence suggests that in cities or districts with Black elected officials, Black citizens are more likely to demonstrate greater levels of political interest and knowledge, participate and vote more frequently, and have higher levels of political trust (Banducci et al. 2004; Bobo and Gilliam 1990; Vanderleeuw and Liu, 2002). Similar results have been found among Māori in New Zealand (Banducci et al. 2004) and Latina/o (Pantoja and Segura 2003) and women voters in the United States (Burns et al. 2001; Liu and Banzszak 2017; Wolbrecht and Campbell 2017) when they are in districts with candidates or representatives who share their identities. However, much of this literature is dated and this subject should be revisited given recent increases in candidate diversity in countries around the world.

Related to these findings is the idea that when members of under-represented groups see few people like themselves in politics, they internalize the idea that politics is not for people like them. This might generate anxiety about political environments and questions about the appropriateness of their involvement within them. This sense of angst about political engagement might be even greater for groups that have traditionally faced high levels of stigmatization and discrimination. This might result in lower levels of interest, knowledge, and self-efficacy as voters turn away from an arena that potentially threatens their self-identity (Horvath 2018).

Much of the research on candidate–voter affinities has focused on the impact of high-profile politicians out of a belief that leaders have a stronger effect on voters than do local candidates.^6^ Still, evidence indicates that local campaigns in Canada are highly personalized affairs (Cross and Young 2015) and that local candidates might be important factors in states with single-member plurality electoral systems (Balmas and Sheafer 2015). Furthermore, research shows that the quality of a local candidate can influence non-partisans and more informed voters (Roy and Alcantara 2015). Local candidates might also seem more approachable and relatable to the average voter than party leaders or elites. Individuals are more likely to know them, or know about them, and thus might feel they have more in common with these candidates than with more elite politicians. It is also possible that voters will identify more strongly with these candidates and their experiences.

To date, little research has actually looked at the role that local candidates, and particularly candidates from under-represented groups, play on the potential for enhanced political engagement. Extant research examines the impact of the overall diversity and number of candidates running in an election on voter political participation, but not the impact of a specific type of candidate on voters in a specific electoral district (Amuedo-Dorantes and Bucheli 2020). The results of these studies indicate that increases in candidate racial diversity in the U.S. are linked to increases in registration and turnout to vote, particularly among Hispanic and younger voters. Research on the potential for enhanced political engagement of 2SLGBTQI+ individuals is non-existent.

### Testing Candidate–Voter Affinities in Canada

Canada presents an important case, both theoretically and pragmatically, to test the impact of 2SLGBTQI+ candidate–voter affinities on voter turnout. First, as one of the most progressive countries in the world in terms of 2SLGBTQI+ rights, Canada enables us to explore these affinities in a country least likely to produce a sense of linked fate threat.^7^ Furthermore, while advocacy organizations attempted to bring more attention to 2SLGBTQI+ issues during the 2021 campaign, parties’ platforms contained few policies specific to their interests and news coverage paid little attention to these policies (Everitt and Wagner 2025). No party, including the right-wing Conservative Party of Canada, was blatantly anti-2SLGBTQI+, although left-wing parties were perceived stronger at promoting queer interests.

More importantly, the number of candidates who publicly identify as 2SLGBTQI+ now means that many 2SLGBTQI+ voters across Canada have such candidates in their district. In the 2021 federal election, 70 candidates were on the ballot in 66 of the 338 federal districts. Four districts had two 2SLGBTQI+ candidates on the ballot. In addition, the country’s parliamentary system and single-member plurality electoral system means that Canadian candidates run as standard bearers for their party in individual districts, not as part of a party list. While party leaders are important to electoral outcomes, it is the local candidates for whom a voter casts their ballot, not the leader. Furthermore, the multi-party system, which regularly sees a minimum of four or five candidates in a district, presents several opportunities for voters to be exposed to 2SLGBTQI+ candidates.

In 2021, eight 2SLGBTQI+ candidates were elected to federal office, an increase from four in the 2019 election. Although a significant proportion of the 70 2SLGBTQI+ candidates ran for the more minor left-leaning parties, including the New Democratic Party (41) and the Green Party (10), the majority of 2SLGBTQI+ voters voted for the centrist Liberal Party, which ran only 12 2SLGBTQI+ candidates, and the right-wing Conservatives, which only ran four. The far right-wing People’s Party of Canada ran one. No publicly out 2SLGBTQI+ individual ran for the Bloc Quebecois, a party that only runs candidates in the province of Quebec, while two others ran as independents or for a fringe party. As might be deduced from their low success rate, many 2SLGBTQI+ candidates lost their campaigns, with a significant proportion running as “sacrificial lambs” in districts where their political party was not competitive (Everitt and Tremblay 2023; Lapointe et al. 2024). While the majority ran in major metropolitan cities, 2SLGBTQI+ candidates also ran in rural communities and mid-sized towns.

A critical advantage that the Canadian case provides is the inclusion of questions in the 2021 Canadian Election Survey (CES) measuring sexual orientation and gender identity. The CES consists of an online campaign-period survey (*N* = 20,968) and an online post-election follow-up survey (*N* = 15,069), where campaign-period respondents were recontacted (Stephenson et al. 2022). Along with its question about sexual orientation, the survey included a non-binary option in its gender identity variable and questions on transgender or Two Spirit identities. These questions can be combined to create a comprehensive 2SLGBTQI+ variable.^8^ Given that the 2SLGBTQI+ population is spread across the country and do not form significant proportions of any given district, the larger sample size from this aggregate identity is important to enable us to quantitatively compare the participation rates of voters in districts with 2SLGBTQI+ candidates to those without such candidates.^9^

Although each candidate included in this study self-identified as 2SLGBTQI+,^10^ we recognize that variation exists in the degree to which their identity was emphasized in their campaigns. Some candidates made it highly visible to voters by wearing rainbow items, having rainbow flags on their Twitter accounts, or speaking openly about their queer identities in their candidate web biographies. Others were listed on the ProudPolitics^11^ website https://www.proudpolitics.org/ or were profiled in stories in 2SLGBTQI+ community newspapers such as *Fugues* and *Xtra* or by more mainstream media outlets such as the Canadian Broadcasting Corporation or local newspapers. Even candidates who were less public about their 2SLGBTQI+ identity were likely to be recognized as such by members of the district’s 2SLGBTQI+ community, as this information was likely shared during a campaign. Nonetheless, we acknowledge that candidates’ messages to their in-group might be a critical mediating factor in mobilization outcomes and intend to consider this in future research examining candidate communications via social media and legacy media platforms.

In this initial analysis, however, we test whether the presence of a local 2SLGBTQI+ candidate is sufficient to increase political turnout among voters in that district. We argue that even if a candidate was not highly vocal about their 2SLGBTQI+ identity or ran for a minor party that received little media attention, many 2SLGBTQI+ voters would still be aware of the candidate’s queer identity, communicated through personal social networks, discussions with other members of the 2SLGBTQI+ community, and even subtle or coded public references to the candidate’s sexual or gender identity.

As past examinations of turnout rates among 2SLGBTQI+ individuals in Canada are limited and contradictory, we approach the data with the following expectations drawn from the comparative literature on 2SLGBTQI+ political participation. Unlike other under-represented individuals who are often disengaged and inhibited from political participation, 2SLGBTQI+ voters appear to be more engaged, possibly due to the desire to protect the rights they have fought hard to achieve. Given this, we anticipate that:

H^1^—2SLGBTQI+ identified voters in Canada will have similar, if not higher, levels of political turnout as other Canadians that persists even with individual-level controls for variables commonly associated with turnout such as age, university-level education, gender, income, and party identification.

Given the potential for differences in the types of districts in which 2SLGBTQI+ and non-2SLGBTQI+ candidates run, we add in district controls including the level of district urbanicity as well as the presence of LGBTQ+ candidates and turnout levels in the district in the previous election.

H^2^—2SLGBTQI+ identified voters in Canada will have similar, if not higher, levels of political turnout as other Canadians that persist even with controls for district-level variables commonly associated with turnout including the level of district urbanicity, previous exposure to LGBTQ+ candidates, and turnout levels in the 2019 election.

Finally, we extend the analysis to explore the impact that candidate–voter affinity might have on turnout rates. Based on evidence of such effects with other minority groups, we hypothesize that:

H^3^—2SLGBTQI+ identified voters who are in districts with a 2SLGBTQI+ candidate are more likely to vote than 2SLGBTQI+ voters in districts with no 2SLGBTQI+ candidate.

While our data do not allow us to attribute motivations to the different results of these analyses, they provide us with opportunities to speculate on the potential usefulness of theories of social identity and linked fate when explaining the results. For example, should H^1^ or H^2^ be supported, we will know that something unique about LGBTQ+ voters leads them to be mobilized at higher levels than other voters with similar individual- or district-level profiles, even in a jurisdiction with few threats to their rights. More importantly, if H^3^ is supported, we might consider looking to theories of group efficacy that suggest the presence of a 2SLGBTQI+ candidate in a 2SLGBTQI+ voter’s constituency might be correlated to reduced feelings of alienation from the political system and a greater likelihood to vote.

## Methods

We study 2SLGBTQI+ political engagement using data from the weighted online post-election wave of the 2021 Canadian Election Study (CES) that included the turnout question (Stephenson et al. 2022).^12^ The study included 1,772 respondents, or 11% of the sample, who self-identify as 2SLGBTQI+. However, only 396 of these voters were in districts with 2SLGBTQI+ candidates. Overall, only 64 respondents identified as transgender, 38 as non-binary or genderqueer, and an additional 52 as Two Spirit, leading us to combine them with those who self-identified as a sexual minority (gay, lesbian, bisexual, queer, or something else) into a variable labeled 2SLGBTQI+. We adopted an approach of collapsing all members of the 2SLGBTQI+ community into the same category for two reasons. First, although not insignificant, the number of transgender, non-binary, and Two Spirit individuals was small (Egan 2025). Second, evidence suggests lesbians, gay men, bisexuals, and other sexual minorities might hold different ideological perspectives than do those who are gender minorities (Albaugh et al. 2024; Guntermann and Beauvais 2022; Perrella et al. 2012, 2019), but we might expect them to respond in similar ways to the experience of having a 2SLGBTQI+ candidate in their district. The shared identity or sense of linked fate that comes from being a traditionally stigmatized minority population is likely to provide an in-group identity that is more cohesive than differentiating (Egan et al. 2008). This is supported by recent research testing LGBTQ coalition identity and linked fate in Canada (Albaugh et al. 2023).

A further reason to rely on the catchall category of 2SLGBTQI+ is that the diversity among the candidates who ran in 2021 is limited. One-fifth (14) of the 70 candidates were transgender, non-binary, or Two Spirit, while the remaining four-fifths were cisgender men (26) and cisgender women (30). More of a challenge is the fact that while some candidates identified as part of the 2SLGBTQI+ community, others were “out” through their identification with their same-sex partners but did not necessarily identify whether they were gay, lesbian, or bisexual. This lack of precision in our measurement of the 2SLGBTQI+ candidates, and the fact that voters’ experience of being a sexual or gender minority is likely to generate affinities with the larger community of 2SLGBTQI+ candidates, accounts for our use of the collective 2SLGBTQI+ identity rather than more specific categories.

The dependent variable in this study is voter turnout measured by a question that read “The federal election was held on Monday, September 20. In any election, some people are not able to vote because they are sick or busy, or for some other reason. Others do not want to vote. Did you vote in the recent federal election?” The response options were yes, no, and a list of other options providing more information on why someone chose not to vote. This was transformed into a dummy variable where those who voted were assigned a 1 and those who did not for whatever reason were assigned a 0. Eighty-nine percent of respondents reported voting, far higher than the 63% official turnout rate. Self-reported turnout is commonly inflated and subject to poor recall, and the CES has been found to produce among the highest gaps (McAllister and Quinlan 2022). However, little reason exists to believe that such bias is more likely to be found among 2SLGBTQI+ respondents than others in the study, and we are concerned only with relative turnout, not absolute levels.

Our primary independent variable is whether voters self-identified as 2SLGBTQI+ or not. As noted above, this variable was developed from variables found in the CES dataset that measured gender identity (man/woman/non-binary/another gender), transgender identity, Two Spirit identity, and voter sexual orientation (heterosexual, homosexual, bisexual, queer, or other sexuality). Likewise, when measuring candidate identity, we collapse the queer sub-identities into the 2SLGBTQI+ group. We then created an interaction variable labeled LGBTQ+ Voter * Candidate to distinguish those 2SLGBTQI+ voters who had 2SLGBTQI+ candidates in their district from those who did not.

Unlike other research, we are less concerned with partisanship affinities in our analysis for several reasons. First, we are not interested in whether a voter is more or less likely to vote for a 2SLGBTQI+ candidate. Our interest lies in whether such a candidate will arouse a sense of interest and engagement in the election that would lead a voter to be more likely to vote in it. While this type of impact might be greatest if the 2SLGBTQI+ candidate is running for the voter’s preferred party, we would argue such engagement is still likely to be stimulated even if the candidate is running for another party. Nonetheless, we developed a variable to measure the existence of a partisan affinity as well as a 2SLGBTQI+ identity affinity (i.e., 2SLGBTQI+ candidates and voters who share a partisan identity).

Other independent control variables included education, age, income, and gender as these are frequently found to be highly correlated with turnout (Blais et al. 2004).^13^ Also included were dummy variables measuring respondents’ party identification as either Liberal, Conservative, or New Democratic, the three major parties in the election and the ones that captured the experiences of most voters. Finally, to test district-level impacts, we included measures for district voter turnout in the previous (2019) election as well as the level of district urbanicity. The first measure came from past Elections Canada information. It should be noted that of the 66 districts with one or more 2SLGBTQI+ candidates in the 2021 election, almost half (30) had a 2SLGBTQI+ candidate in the 2019 election. As a result, we also included a variable indicating whether a 2SLGBTQI+ candidate had run in this district in the previous election. The level of district urbanicity information came from a measure created by Armstrong and his colleagues (2022) that captures multiple dimensions of urbanization to produce a scale that ranges from +1.49 for the most urban district to −1.44 for the most rural district.

The models are multi-level logistic regression models. Given Canada’s electoral system, survey respondents are clustered within electoral districts: candidates run in a specific district, and voting occurs within this local context. Thus, we tested the need to account for district-level variation. The Intraclass Correlation Coefficient is a moderate value of 0.08, indicating that eight percent of variation in turnout is entirely due to district differences. A Likelihood Ratio test confirms that the fit of a model with a random intercept by district is significantly better than a fixed-effects only model (χ^2^(1) = 1044.5, *p* = 0.00).

## Analysis

We begin by comparing the turnout rates of sexual and gender minorities to other Canadians. This enables us to test our first hypothesis and determine whether being 2SLGBTQI+ has an impact on voter turnout, like it does in other parts of the world (Turnbull-Dugarte and Townsley 2020). Model 1 in Table 1 shows that when 2SLGBTQI+ identity is included along with common socio-demographic and partisan controls in a multi-level logistic regression equation for turnout, the coefficient appears substantive and statistically significant, supporting our expectations in H^1^. University-level education, age, and income were also positively correlated, while being a woman was negatively correlated. The measures of Liberal, Conservative, and NDP partisan identities also proved statistically significant and help control for the possibility that higher levels of turnout among 2SLGBTQI+ voters were linked to the larger number of NDP respondents with such candidates in their district. These results suggest that, unlike the findings of Perrella and his colleagues (2019), 2SLGBTQI+ voters in Canada match those in other countries in terms of predisposition to vote.

**Table 1.** Impact of Candidate—Voter Affinities on Turnout.

	2SLGBTQI+ Turnout (1)	2SLGBTQI+ Turnout w/ District-Level Variables (2)	2SLGBTQI+ Voter * Candidate (3)	2SLGBTQI+ Voter * Candidate w/District-Level Variables (4)
2SLGBTQI+ voter	0.26^***^	0.27^***^	0.11	0.10
	(0.08)	(0.08)	(0.09)	(0.09)
2SLGBTQI+ candidate			−0.10	−0.10
			(0.10)	(0.10)
University education	0.69^***^	0.69^***^	0.70^***^	0.69^***^
	(0.07)	(0.07)	(0.07)	(0.07)
Age	0.03^***^	0.03^***^	0.03^***^	0.03^***^
	(0.002)	(0.002)	(0.002)	(0.002)
Income: Medium	0.32^***^	0.31^***^	0.32^***^	0.31^***^
	(0.06)	(0.06)	(0.06)	(0.06)
Income: High	0.67^***^	0.67^***^	0.67^***^	0.66^***^
	(0.07)	(0.07)	(0.07)	(0.07)
Gender identity: Woman	−0.05	−0.04	−0.04	−0.04
	(0.05)	(0.05)	(0.05)	(0.05)
Gender identity: Non-binary	0.09	0.06	0.13	0.29
	(0.62)	(0.63)	(0.63)	(0.65)
Gender identity: Other	−0.18	−0.14	−0.12	−0.09
	(1.03)	(1.03)	(1.03)	(1.04)
Party ID: Liberal	1.27^***^	1.28^***^	1.28^***^	1.28^***^
	(0.07)	(0.07)	(0.07)	(0.07)
Party ID: Conservative	1.41^***^	1.40^***^	1.40^***^	1.39^***^
	(0.08)	(0.08)	(0.08)	(0.08)
Party ID: NDP	1.44^***^	1.44^***^	1.43^***^	1.42^***^
	(0.08)	(0.08)	(0.08)	(0.08)
Party ID: Other	1.19^***^	1.18^***^	1.19^***^	1.17^***^
	(0.09)	(0.09)	(0.09)	(0.09)
District: Turnout		0.03^***^		0.02^***^
		(0.01)		(0.01)
District: Urbanicity		0.02		0.02
		(0.05)		(0.05)
Previous 2SLGBTQI+ candidate (2019 election)		−0.05		−0.05
		(0.09)		(0.10)
2SLGBTQI+ voter * candidate			0.83^***^	0.81^***^
			(0.20)	(0.20)
Intercept	−0.86^***^	−2.53^***^	−0.84^***^	−2.45^***^
	(0.11)	(0.51)	(0.11)	(0.51)
Random effects (variance)				
District intercept	0.25	0.24	0.26	0.24
*N*	15,684	15,499	15,499	15,499
Pseudo-R^2^ FE/Total	0.18/0.24	0.18/0.24	0.18/0.24	0.18/0.24

^*^p < .1; ^**^p < .05; ^***^p < .01.

We next consider how these findings are affected by district-level variables. The results, as presented in Model 2 in the second column of Table 1, suggest clear support for H^2^ that differences in the turnout rates of 2SLGBTQI+ persist regardless of the type of district in which they are located. Neither the degree of urbanicity or exposure to a previous 2SLGBTQI+ candidate reached statistical significance in these models and past turnout rates had only a small effect.

**Figure 1.**
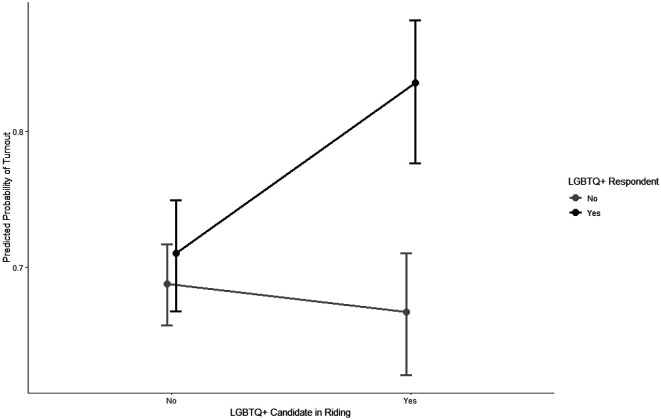
Interaction plot for respondent * candidate for turnout. Note: The interaction plot in Figure 1 demonstrates no difference in the turnout rate for 2SLGBTQI+ respondents and heterosexual Canadians when no 2SLGBTQI+ candidate is in their district and a substantial and significant difference when there is.

Finally, we introduce exposure to a current 2SLGBTQI+ candidate into our models. Figure 1 plots the predicted probability of turnout and the interaction between 2SLGBTQI+ voters and the presence of a 2SLGBTQI+ candidate in their district. The results support H^3^ in that 2SLGBTQI+ identified voters who are in districts with a 2SLGBTQI+ candidate are much more likely to vote than those in districts with no 2SLGBTQI+ candidate; the gap is about 0.12 points. This relationship is also demonstrated by Model 3 in the third column in Table 1, which includes a measure for 2SLGBTQI+ candidates as well as a voter-candidate interaction measure. Model 4 repeats this analysis with the district-level variables. In both models the interaction between 2SLGBTQI+ voters and candidates appears robust and statistically significant. Clearly, having a 2SLGBTQI+ candidate in their district is positively correlated to a 2SLGBTQI+ voter turning out to vote.

To be thorough, we ran additional models not presented here to determine whether turnout was related to the general competitiveness of the district or to partisan affinities between candidates and voters. The results were substantively and statistically similar to the presented models, and none of our core conclusions changed. Finally, as a further test of the impact of affinity, we ran similar models using measures of interest in the election^14^ and political efficacy as the dependent variables^15^ (see Table A2 and A3 in the Appendix). These tests produced slightly mixed results. In both cases, the 2SLGBTQI+ voters were more interested and efficacious than the non-2SLGBTQI+ voters. However, it was only in the model for interest in the election that the interaction variable for 2SLGBTQI+ Voter*Candidate reached statistical significance. This result suggests that, as with turnout, voters who live in districts with a 2SLGBTQI+ candidate (regardless of voter/candidate party affinities) demonstrate higher levels of interest than 2SLGBTQI+ voters in districts without such candidates. Political efficacy, however, had no measurable impact. Upon reflection, we conclude that efficacy as measured in the CES is a more deep-seated and general assessment of government and its relationship with citizens than things like political interest or decision to vote. It might thus be less responsive to the presence of a specific type of (local) candidate in one election.

## Discussion

Our results reveal that the turnout of 2SLGBTQI+ voters in Canada is typically higher than that of heterosexual Canadians, matching that of similar voters elsewhere in the world (Turnbull-Dugarte and Townsley 2020). Even with controls for common correlates with turnout, these analyses indicate that 2SLGBTQI+ voters are actually more politically engaged than the non-2SLGBTQI+ population and that these higher turnout rates are positively correlated with candidate–voter affinities. When located in districts with 2SLGBTQI+ candidates, 2SLGBTQI+ voters are more likely than non-2SLGBTQI+ voters to cast a ballot in the election. Those 2SLGBTQI+ voters without such candidates in their districts vote at similar rates as non-2SLGBTQI+ voters. This suggests that affinities can have an independent and positive effect on political engagement, potentially empowering voters who are systematically under-represented in politics to become more engaged in an election campaign regardless of whether they intend to vote for an affinity candidate or not. Such descriptive representation among the candidates might have the effect of reducing the sense that politics is not for people like them despite past experiences of stigmatization and discrimination. In other words, increases in the number of “identity” candidates might play an important role in offsetting the democratic disengagement and declining turnout rates among voters in western industrialized societies. This argument is supported by similar results produced for other measures of political engagement, including interest in election campaigns.

Linked to this is the recognition that Canadian 2SLGBTQI+ voters do not appear to be as highly mobilized as 2SLGBTQI+ voters in other political jurisdictions. They are not demonstrably more likely to vote than the average Canadian unless they have a 2SLGBTQI+ candidate in their district to stimulate their sense of political engagement. These results help us to better understand the strength of competing arguments presented to account for candidate–voter affinities. For example, in Canada, turnout appears to be the strongest when 2SLGBTQI+ voters are presented with 2SLGBTQI+ candidates as political options. This supports arguments that these affinities are linked to explanations of empowerment rather than linked fate. It mattered little that 2SLGBTQI+ voters were Conservative, Liberal, or NDP partisans or that the 2SLGBTQI+ candidates were running for any of these parties. Voters were not more likely to vote for NDP candidates, who they might have thought would be better at protecting their interests because of that party’s traditional support for the 2SLGBTQI+ community. Instead, it was the simple presence of a 2SLGBTQI+ candidate from any party running in the district that was correlated with higher 2SLGBTQI+ voter turnout.

The limited impact of the linked fate explanation might be the result of the context of 2SLGBTQI+ politics in Canada. Our results suggest that Turnbull-Dugarte and Townsley’s (2020) argument that 2SLGBTQI+ individuals over participated in politics as a way to protect their rights from being overturned might be less relevant in a country like Canada, which has enacted legal protections for this community and has a weak social conservative element to threaten movement gains. It might, however, still be relevant in countries where the most egregious of forms of social exclusion and oppression have not yet been eliminated, or in future Canadian elections should the growing pushback against 2SLGBTQI+ rights appearing elsewhere begin to occur here as well.

In exploring the under researched field of candidate–voter affinities, this paper is unique in its effort to tie together the growing literature on 2SLGBTQI+ candidates and voter behavior. Clearly, more work needs to be done to tease out the nuances of these affinities. For example, questions arise about the mediating role of candidate self-presentation. How does the way a candidate employs their identity factor into this relationship? We assumed going into this analysis that voters would be aware of the sexual orientation and gender identity of the 2SLGBTQI+ candidates in their districts. However, some candidates might have actively highlighted their identity and had this widely acknowledged in news coverage while others might have been less visible to their potential voters (Wagner 2019). A more thorough examination of the manner in which 2SLGBTQI+ candidates communicated their identity and thereby triggered its importance to voters might be a necessary intervening step before fully assessing these affinities.

All in all, our findings suggest that candidate–voter affinities are an important factor to consider when thinking about political engagement. More importantly, they have significant implications for electoral democracy and voter turnout, particularly in environments where turnout has declined and citizens are increasingly disengaged and distrustful of their governments and politicians.

## Supplemental Material

Supplemental Material - Examining the Effects of 2SLGBTQI+ Candidates on 2SLGBTQI+ Voter Turnout in Canada Supplemental Material for Examining the Effects of 2SLGBTQI+ Candidates on 2SLGBTQI+ Voter Turnout in Canada by Joanna Everitt, Kenny William Ie, Karen Bird, Angelia Wagner, and Mireille Lalancette in Political Research Quarterly.

## Notes

1. See the special issue on “Research on Race and Ethnicity in Legislative Studies” in *PS: Political Science and Politics* Vol 55:2, 2022.

2. We use the acronym 2SLGBTQI+ to refer to individuals who self-identify as Two Spirit, lesbian, gay, bisexual, transgender, or another minority sexual identity such as asexual or pansexual. This is the acronym used by Statistics Canada to be most inclusionary (https://www.canada.ca/en/women-gender-equality/free-to-be-me/2slgbtqi-plus-glossary.html).

3. Questions measuring sexual orientation first appeared in the Canadian Election Study in 2019 but had previously been included in the Canadian General Social Survey in 2008 and 2013. It was not until 2019 that the Canadian Election Study offered “other” (e.g., trans, non-binary, Two Spirit, and genderqueer) as an option in the question assessing voters’ sex, although Albaugh and colleagues (2023) note issues with the way this question is worded. Canada is not unique. While the American National Election Study included questions about respondents’ sexual orientation since the late 1990s, it first included an “other” option to the question measuring sex in 2016. Few European election studies had included questions about sexual orientation by the late 2010s, nor had they measures that provided the choice of “other” when asked to identify a respondent’s sex. This dramatically limits our ability to examine historical patterns of queer participation.

4. Recent opposition to trans rights was mobilized in the period after the 2021 election.

5. These data are available at the University of New Brunswick Data Repository upon request to the corresponding author.

6. See a recent paper by Albaugh and Baisley (2023) that identifies voter affinity effects in Ontario when Kathleen Wynne, lesbian leader of the Liberal Party, ran in the 2014 provincial election.

7. See work by Kamenou (2024) to understand the motivations for turnout in a country that is politically hostile to 2SLGBTQI+ individuals.

8. The gender question asked “Are you ….?” and then provides several response options: A man, A woman, Non-binary, Another gender, please specify, with a follow-up text box. Another question asked: “Are you transgender? (Yes, No, Don’t Know/Prefer not to say). At the end of the campaign wave survey were the questions “Are you Two Spirit?” (Yes, No, I don’t Know/Prefer not to answer) and “Which of the following best represents how you think of yourself?” with options Straight or heterosexual, Gay or Lesbian, Bisexual, Queer, Something else, I am not sure yet, I don’t know what this question means, and Prefer not to answer.

9. Due to deliberate oversampling in districts with identified gay villages, this election survey likely had a larger percentage of 2SLGBTQI+ respondents than previous ones. The use of survey weights and multi-level modeling has corrected for this unrepresentativeness.

10. This information comes from a database of all *out* 2SLGBTQI+ candidates in Canadian federal and provincial elections created by Joanna Everitt and updated after each election. Information is drawn from official candidate biographies, websites of queer advocacy organizations like ProudPolitics, election coverage produced by mainstream and queer news outlets, and general web searches. It is regularly compared to that produced by organizations and other academics working in this field. While this dataset may not have captured all 2SLGBTQI+ candidates in all parts of the country, it is the most complete record we have.

11. ProudPolitics is an organization supporting 2SLGBTQI+ candidates running for public office.

12. The data were weighted using the pes21_weight_general_all variable that came with the CES dataset and was created using an iterative “raking” process, as provided by the ipfraking command in STATA15. Marginal values were successively weighted according to province, as well as gender, age group, and education level (Stephenson et al. 2022).

13. Summary statistics for these independent variables are available in Table A1 in the Appendix.

14. Interest was measured with the question “How interested are you in this federal election? Set the slider to a number from 0 to 10, where 0 means no interest at all, and 10 means a great deal of interest.”

15. Efficacy was measured using the questions “Sometimes, politics and government seem so complicated that a person like me can’t really understand what’s going on”; “People like me don’t have any say about what the government does”; and “The government does not care much about what people like me think.” We ran tests for efficacy using each question individually and all three combined into an efficacy scale.
