# Supplementary material for: Examining the Effects of 2SLGBTQI+ Candidates on 2SLGBTQI+ Voter Turnout in Canada
Source: Polit Res Q. 2025 Jun 26;78(4):1273–85. doi: 10.1177/10659129251355606 (PMC12527295; doi:10.1177/10659129251355606)
Supplement: Supplemental Material - Examining the Effects of 2SLGBTQI+ Candidates on 2SLGBTQI+ Voter Turnout in Canada [file sj-pdf-1-prq-10.1177_10659129251355606.pdf]

Appendix

Table A1 - Summary Statistics Table

	N	Mean	SD	Min.	Max
Turnout (0/1)	15,731	0.89	0.31	0	1
Interest in Election (0-1)	15,532	0.67	0.26	0	1
Efficacy (0-1)	15,012	0.42	0.23	0	1
2SLGBTQI+ Voter (0/1)	15,731	0.11	0.31	0	1
2SLGBTQI+ Candidate (0/1)	15,546	0.22	0.41	0	1
University Education (0/1)	15,731	0.54	0.50	0	1
Age (Years)	15,731	53.27	16.70	18	96
District: Turnout (%)	15,546	67.26	5.07	46.40	79.00
District: Urbanicity	15,546	0.53	0.75	-1.44	1.49
Previous (2019) 2SLGBTQI+ Candidate (0/1)	15,546	0.24	0.43	0	1
Income	15,731	Low: 5,684 (36.13%)			
		Medium: 4,845 (30.80%)			
		High: 5,202 (33.07%)			
Gender identity	15,731	Man: 7,293 (46.36%)			
		Woman: 8,369 (53.20%)			
		Non-binary: 44 (0.28%)			
		Other: 25 (0.15%)			
Party ID	15,731	Liberal: 4,881 (31.03%)			
		Conservative: 3,722 (23.67%)			
		NDP: 2,191 (13.93%)			
		Other: 2,100 (13.35%)			
		None: 2,837 (18.03%)			

Table A2 – Impact of Candidate - Voter Affinities on Interest in the Election

	2SLGBTQI+ Interest (1)	2SLGBTQI+ Interest w/ District-level Variables (2)	2SLGBTQI+ Voter * Candidate (3)	2SLGBTQI+ Voter * Candidate w/ District-level Variables (4)

2SLGBTQI+ Voter	0.04***	0.04***	0.03***	0.03***
	(0.01)	(0.01)	(0.01)	(0.01)
2SLGBTQI+ Candidate			-0.002	-0.004
			(0.01)	(0.01)
University Education	0.04***	0.04***	0.04***	0.04***
	(0.005)	(0.005)	(0.005)	(0.005)
Age	0.003***	0.003***	0.003***	0.003***
	(0.0001)	(0.0001)	(0.0001)	(0.0001)
Income: Medium	0.03***	0.03***	0.03***	0.03***
	(0.005)	(0.005)	(0.005)	(0.005)
Income: High	0.02***	0.02***	0.02***	0.02***
	(0.01)	(0.01)	(0.01)	(0.01)
Gender identity: Woman	-0.04***	-0.04***	-0.04***	-0.04***
	(0.004)	(0.004)	(0.004)	(0.004)
Gender identity: Non-binary	0.05	0.05	0.06	0.06
	(0.05)	(0.05)	(0.05)	(0.05)
Gender identity: Other	-0.07	-0.06	-0.06	-0.06
	(0.07)	(0.07)	(0.07)	(0.07)
Party ID: Liberal	0.19***	0.19***	0.19***	0.19***
	(0.01)	(0.01)	(0.01)	(0.01)
Party ID: Conservative	0.21***	0.21***	0.21***	0.21***
	(0.01)	(0.01)	(0.01)	(0.01)
Party ID: NDP	0.19***	0.19***	0.19***	0.19***
	(0.01)	(0.01)	(0.01)	(0.01)
Party ID: Other	0.15***	0.15***	0.15***	0.15***
	(0.01)	(0.01)	(0.01)	(0.01)
District: Turnout		0.001**		0.001**
		(0.001)		(0.001)
District: Urbanicity		0.01**		0.01**
		(0.004)		(0.004)
Previous 2SLGBTQI+ candidate (2019 election)		0.003		0.002
		(0.01)		(0.01)

2SLGBTQI+ Voter * Candidate			0.05^{***}	0.05^{***}
			(0.01)	(0.01)
Intercept	0.35^{***}	0.25^{***}	0.35^{***}	0.26^{***}
	(0.01)	(0.04)	(0.01)	(0.04)
Random Effects (Variance)				
District Intercept	0.00	0.00	0.00	0.00
N	15,486	15,308	15,308	15,308
Pseudo-R^2 FE / Total	0.13/0.17	0.14/0.17	0.13/0.17	0.14/0.17

$^{*}p < .1$; $^{**}p < .05$; $^{***}p < .01$

The Interest question asked “How interested are you in this federal election? Set the slider to a number from 0 to 10, where 0 means no interest at all, and 10 means a great deal of interest.” It was recoded to 0-1.

Figure A1 Interaction Plot for Respondent * Candidate for Interest in the election

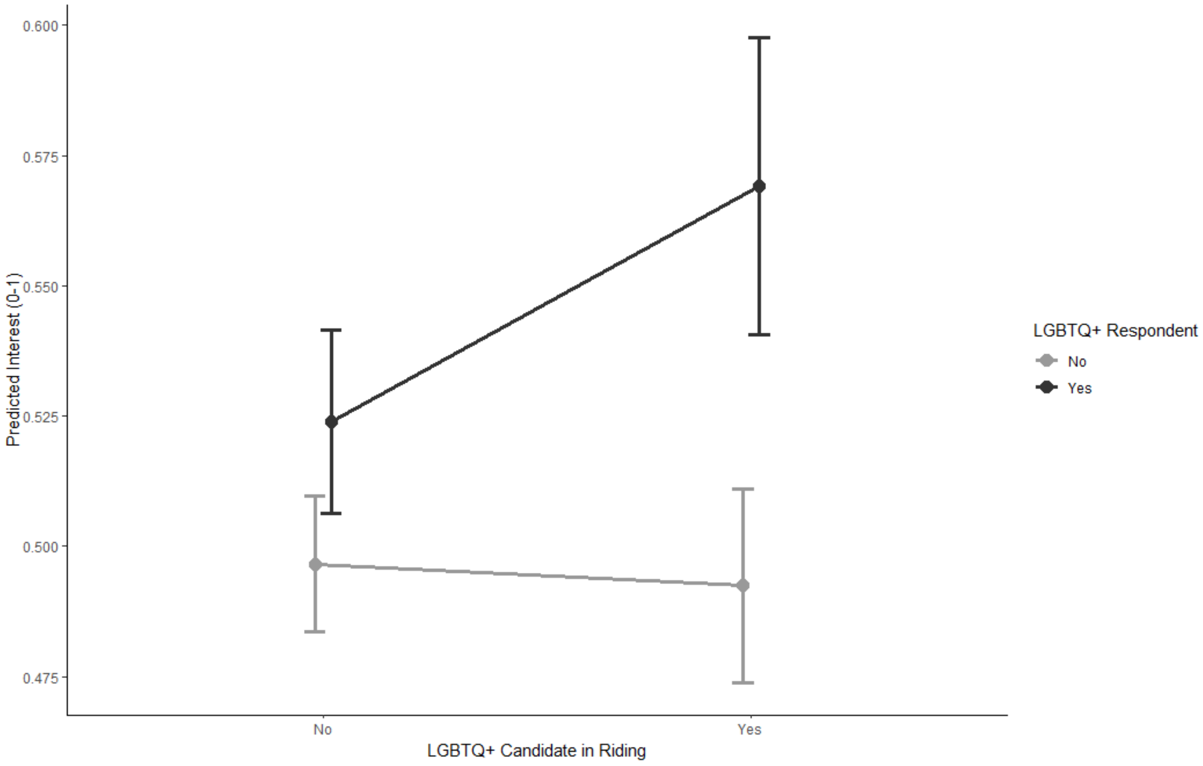

Figure Caption – The interaction plot demonstrates no difference in the level of interest in the election for 2SLGBTQI+ respondents and heterosexual Canadians when there is no 2SLGBTQI+ candidate in their district and substantial and significant differences when there is.

Table A3 – Impact of Candidate - Voter Affinities on Voter Efficacy

	2SLGBTQI+ Efficacy (1)	2SLGBTQI+ Efficacy w/ District-level Variables (2)	2SLGBTQI+ Voter * Candidate (3)	2SLGBTQI+ Voter * Candidate w/ District-level Variables (4)
2SLGBTQI+ Voter	0.02***	0.02***	0.03***	0.03***
	(0.01)	(0.01)	(0.01)	(0.01)
2SLGBTQI+ Candidate			-0.001	0.002
			(0.01)	(0.01)
University Education	0.07***	0.07***	0.07***	0.07***
	(0.004)	(0.004)	(0.004)	(0.004)
Age	0.001***	0.001***	0.001***	0.001***
	(0.0001)	(0.0001)	(0.0001)	(0.0001)
Income: Medium	0.03***	0.03***	0.03***	0.03***
	(0.004)	(0.004)	(0.004)	(0.004)
Income: High	0.06***	0.06***	0.06***	0.06***
	(0.004)	(0.004)	(0.004)	(0.004)
Gender identity: Woman	-0.03***	-0.03***	-0.03***	-0.03***
	(0.004)	(0.004)	(0.004)	(0.004)
Gender identity: Non-binary	-0.02	-0.02	-0.02	-0.02
	(0.04)	(0.04)	(0.04)	(0.04)
Gender identity: Other	-0.07	-0.07	-0.07	-0.07
	(0.06)	(0.06)	(0.06)	(0.06)
Party ID: Liberal	0.12***	0.12***	0.12***	0.12***
	(0.01)	(0.01)	(0.01)	(0.01)
Party ID: Conservative	0.02***	0.02***	0.02***	0.02***
	(0.01)	(0.01)	(0.01)	(0.01)
Party ID: NDP	0.06***	0.06***	0.06***	0.06***
	(0.01)	(0.01)	(0.01)	(0.01)
Party ID: Other	0.08***	0.08***	0.08***	0.08***
	(0.01)	(0.01)	(0.01)	(0.01)
District: Turnout		0.0000		0.0000
		(0.001)		(0.001)

District: Urbanicity		0.01		0.01
		(0.005)		(0.005)
Previous 2SLGBTQI+ candidate (2019 election)		-0.01		-0.01
		(0.01)		(0.01)
2SLGBTQI+ Voter * Candidate			-0.002	-0.002
			(0.01)	(0.01)
Intercept	0.25***	0.25***	0.25***	0.25***
	(0.01)	(0.05)	(0.01)	(0.05)
Random Effects (Variance)				
District Intercept	0.00	0.00	0.00	0.00
N	14,966	14,798	14,798	14,798
Pseudo-R^2 FE / Total	0.11/0.17	0.11/0.17	0.11/0.17	0.11/0.17

*p < .1; **p < .05; ***p < .01

The Efficacy Index was based on three measures: “Sometimes, politics and government seem so complicated that a person like me can't really understand what's going on”; “People like me don't have any say about what the government does”; “The government does not care much about what people like me think.”. Variables were reverse-coded so that higher values indicate higher efficacy and re-scaled 0 to 1.
